# A little bit of Africa in Brazil: ethnobiology experiences in the field of Afro-Brazilian religions

**DOI:** 10.1186/1746-4269-10-12

**Published:** 2014-01-27

**Authors:** Ulysses Paulino Albuquerque

**Affiliations:** 1 Departamento de Biologia, Área de Botânica, Laboratório de Etnobiologia Aplicada e Teórica (LEA), Universidade Federal Rural de Pernambuco, Rua Dom Manoel de Medeiros s/n, Dois Irmãos, Recife 52171-030, Pernambuco, Brasil

**Keywords:** Ethnobiology, Ethnobotany, Ethnography, Fieldwork, Evocations

## Abstract

This essay, which is the fourth in the series “Recollections, Reflections, and Revelations: Ethnobiologists and Their First Time in the Field”, is a personal reflection by the researcher on his first field experience with ethnobiology of so called Afro-brazilian cults. The author recounts his feelings and concerns associated with initial fieldwork.

## My initiation into ethnobiology

I was completely captivated by the activity proposal presented to me by my professor of systematic of phanerogams^a^, Dr. Laise de Holanda Cavalcanti Andrade, at the Universidade Federal de Pernambuco. As a BA student near completion in biological sciences, I had devoted my entire attention to marine biology. It never occurred to me, even for a second, to study plants or anything related to them. Nevertheless, this proposal seemed curious. It was studying not only plants but also their relationship with people. This seemed both different and fascinating. The term “ethnobotany” appeared in the many conversations held to clarify the work that needed to be done to complete the course. The challenge was even greater when the teacher concluded that the study should address the plants used in Afro-Brazilian cults.

The study did not require any fieldwork, but I could not miss the opportunity to venture into areas outside the university and, even more, to venture into the universe of “African magic”. Although I was quite taken with enthusiasm, my colleagues considered the choice of subject unusual, to say the least. First, because of my complete lack of motivation for studies related to botany and second, because I would be venturing into the “mysterious” Afro-Brazilian religious landscape, which still suffers strong social discrimination. The Afro-Brazilian cults originated in the colonial Brazilian context, where many Africans were brought into conditions of absolute servitude. Forbidden to worship their gods openly, they practiced their rituals in secrecy. Obviously, complete uniformity does not exist in such practices. Depending on the African tribal origins of the Africans who arrived in Brazil’s cities, the practices, rituals and deities are somewhat different.

In modern urban Brazil, these religious practices were subject to police harassment, especially during the government of President Getúlio Vargas in the 1930's^b^. Faced with this reality, the cults practiced in secret and in the poorest areas of the city. Furthermore, people who practiced one of these religions would not openly admit it. At the time, the belief that the Afro-Brazilian religions consisted of “demonic” practices, the worship of different deities, and the ritual sacrifice of animals began to feed the popular imagination. The Afro-Brazilian cults are, in essence, possession cults. The devotees enter a sort of trance to lend their bodies to the manifestation of the deities worshiped. To complete this unfavorable image, a famous and important physician and anthropologist, in the century XIX, Raimundo Nina Rodrigues, treated the trance as a kind of mental disorder^c^.

Well, that was the scenario I faced. I would be facing not only a difficult subject, but also the natural fear of the supporters of Afro-Brazilian cults, who might perceive me as a threat. I was sure of what I wanted, but not how I was going to achieve it. In Afro-Brazilian cults, plants and animals serve an essential, if not irreplaceable, function. There is even an expression in the Yorùbá language that says, “*Kossi Ewe, Kossi Orisha*”, which means “without leaves, no deities”. All the deities of the Afro-Brazilian pantheon have their own plants, which devotees use to prepare medicines, ritual baths, and incense smokers. If every deity has its own plants, we can identify a system of classification of these [1-6]. Such a system allows the plant world to be organized and guides the priests in their practices.

**Figure 1:**
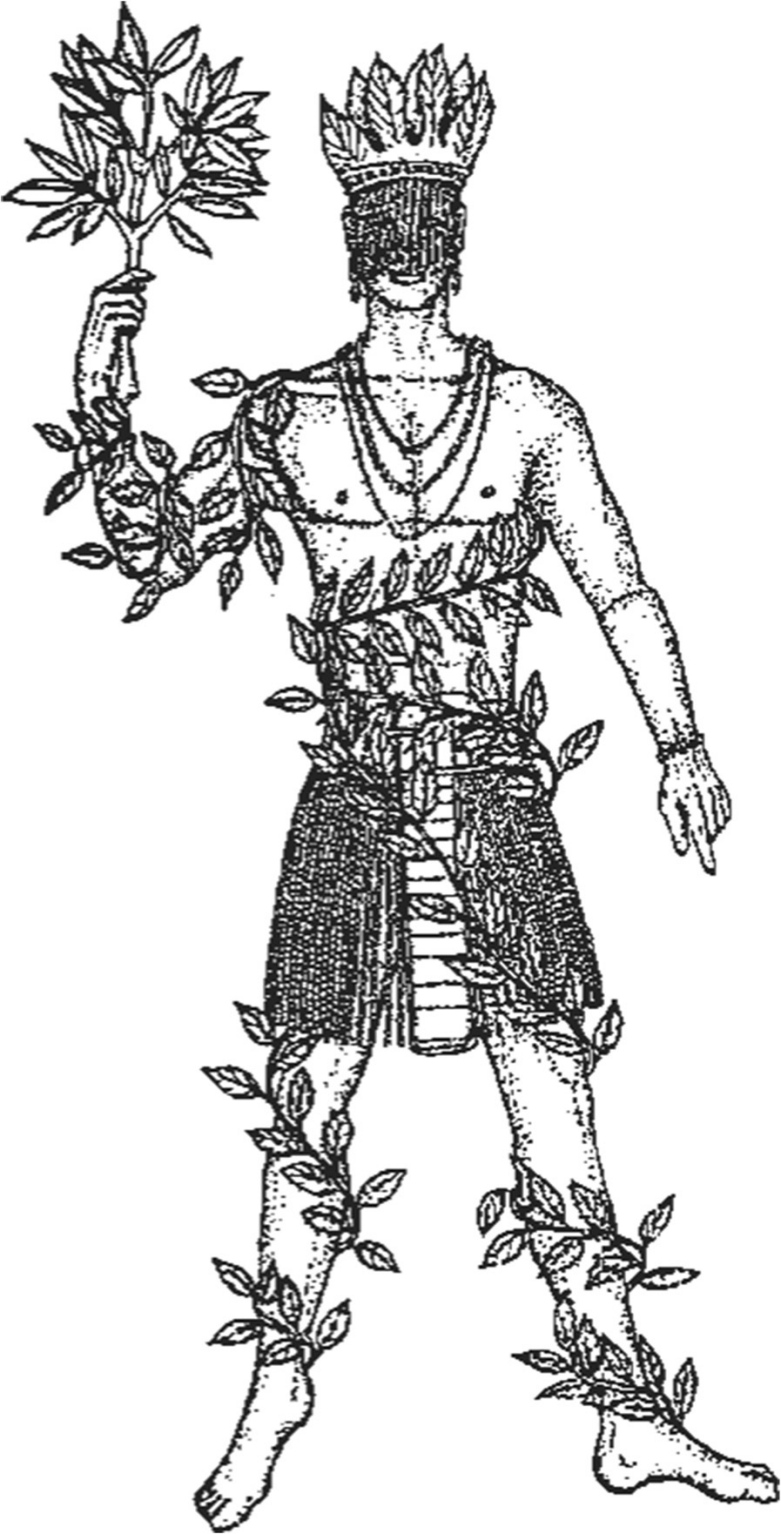
**Free representation of Osanyin.** By Karin Schmalz Peixoto Reproduced from Albuquerque [4].

There is a myth that explains this very well. Osanyin, the deity responsible for the use of plants and their secrets (Figure 1), kept all the “leaves” (= plants) in a container hanging from a tree. The other deities were very jealous of Osanyin because every time they needed to use plants, they had to appeal to him. Some of the deities complained to Yansan, deity of the winds and the guide of the spirits of the dead people (*eguns*). Yansan shook her skirt and made the wind blow so strongly that it scattered all of Osanyin’s leaves. The deities, called *Orishas (Orixás in Portuguese)* in Brazil, rushed to get their own leaves. As they touched the leaves, they imprinted in them their essence and vitalizing energy (the *axé*). However, despite this effort, the *Orishas* have continued to rely on Osanyin because although they have their own plants, Osanyin is the only one who knows how to use them.

In the Afro-Brazilian cults, the Osanyin priest is called *Babalosanyin* (if male) or *Yalorisanyin* (if female). Nowadays, it is more common the main head of the cult is the *Babalorisha* or *Yalorisha*, who handles the sacred plants. In Recife (Pernambuco state) in northeastern Brazil, where I would do my research, the house in which the Afro-Brazilian worship takes place is called *terreiro* (yard). Even the cult has two names, one typically regional (*Xangô (Shango),* the name of one of the deities) and one national (*Candomblé*). Each *terreiro* has autonomy and creates a hierarchy around the figure of the *Babalorisha* or *Yalorisha*.

I recognized the nuances of the world that I was about to enter. Everything in *Candomblé* is sacred and has power: the water, the minerals, the plants, and the animals, as they guard the vitalizing energy of the deities. The spooked word has power. Words are used to sing the special songs that make a medicine work, a magical procedure. Words make the energy trapped in animate and inanimate objects move and expand to the ends desired.

## The first entrance into the field

Having read all the theoretical studies on the subject, especially ones of the most important by the Frenchmen Roger Bastide [7] and Pierre Verger [8], I started questioning myself about my first visit to a *terreiro* of *Candomblé*. I had intended to conduct interviews using questionnaires around simple and direct questions: which plants are used and for what purposes. As a neophyte on the subject, it seemed a methodologically reasonable way to start. However, my readings indicated a need for more qualitative participant observation, the main methodological approach of anthropologists in Brazil. This involved immersion in the world of *Candomblé*, but I was not prepared for this confrontation, although I desired it.

I managed to schedule an appointment with a *babalorisha* who had his *terreiro* established in the Água Fria neighborhood. When it comes to *Candomblé*, one cannot overlook the so-called *Terreiro do Pai Adão* (Father Adão yard), one of the oldest in operation, which was functioning in the same neighborhood. This *terreiro* was led by Father Adão, a famous priest portrayed by many scholars as having an almost mystical aura. The famous Brazilian sociologist Gilberto Freyre [9], author of the well-known book *The Masters and the Slaves (Casa Grande & Senzala)*, wrote of Father Adão and of a sacred tree that he cultivated in his *terreiro*:

One of them, some old strangler fig^d^ of Fundão, the site of the old *babalorixá* father Adão, already dead, an almost giant black, who studied the dark arts in Africa although born and raised in Pernambuco, and mulatto as only he was (…) Seeing this old black giant dancing was a surprise: at dawn he seemed not himself but something as an elf with wings on his feet. They say it was through the magic strangler fig that he communicated with Mother Africa, hearing voices telling him in *nagô*^e^, “Adão, do this. Adão, do that” (…).

I then decided to enter this magical landscape, portrayed by the magic pen of Brazilian anthropologists and sociologists. Following directions, I arrived at a *terreiro* not far from the main access roads. Upon entering, I felt all the grandeur and religiousness emanating from the space. There was a large garden with luxuriant plants and a multitude of herbs. I immediately thought these were plants used in liturgical and medicinal practices. I also noticed that in many cases, the distinction between ritual and medicinal use makes no sense. In *Candomblé,* the ritual is done to bring health and wellness, and a use that in our classification would appear to be purely magical was seen as therapeutic.

After a few more steps, I glimpsed a large hall adorned with large pieces of white cloth, statues of deities, some drums and ritual objects. It was in that space that the public worship took place, where the deities were invoked. To help the non-Brazilian reader understand a bit more, think of the movie *The Serpent and the Rainbow*. The movie was inspired by the book of the same name on the cults of Vodun in Haiti by the ethnobiologist Wade Davis^f^. Removing all the excess of the Hollywood drama, we have something similar: a large hall where people dance to the beat of drums.

I was invited to sit, but I was shy, suspicious, and reserved. I did not know where to start. I asked the first question in an almost inaudible voice, and my first answer came, the name of a plant and its use in ritual. I began to be hopeful, but I realized that my interviewee would not allow himself to be interviewed, because he spoke of things that I did not ask. I soon realized that my interview plan did not fit and that my questions were meaningless for my interviewee. For him, talking about plants only made sense in context. This context might be a description of a ritual, the trivial events of everyday life, or his childhood, when the *Orishas* called him by his name and he had to answer the call to become an initiate in the Afro-Brazilian beliefs. Based on the manuals I had read and on conversations with some people, I expected that the interview would not last more than three hours. I stayed with my first interviewee for fourteen hours. I had the distinct feeling that if he had no ritual obligations to fulfill he would not have let me leave until he had told me all that he knew, or at least all that he could tell.

The plants are one of the great secrets of *Candomblé* (see [10]). The priests conscientiously maintain the information about the sacred plants, so I knew I could not learn everything, much less in one interview. I knew some of the plants that were mentioned because they were grown in the yard. Others could only be collected by the priest, especially when they were intended for the cult initiation rituals, whereas others could be purchased at local markets or fairs. I realized that to understand a little of this dynamic, I needed to visit the markets. I came out of the interview dazed and confused and, of course, with fourteen hours of recordings, many notes in an improvised field notebook, and one certainty: getting out in the field is always a surprise and a unique experience, even with all the background that the best literature on methods can offer. I did not know how to organize that much information, nor was I prepared for it. In my mind, I would fill in a questionnaire with the answers from my informant, thank him for his availability, and leave for another interview.

I was completely saturated with fantasy after this first experience. The world of plants in *Candomblé* was surrounded by many words in the *Yorùbá* language, areas in the *terreiro* where special rituals took place, the deities with their plants, and taboos that I needed to respect to be heard and well received rather than sent off to the sound of curses in a strange language. I went home convinced that I could no longer use the structure that I had planned to, that my interviewees needed to express themselves, and that if I really wanted to understand the relationship between people and plants I needed to participate in the rituals. The mere mention of participating in the rituals to my fellow students made their hair rise, first out of fear, and then out of prejudice. However, that did not matter, because it was necessary.

## The market: space of continuity with the sacred

Visiting the market emerged as an imperative after that first entry into the field. The market, I was told, was central to the sacredness of *Candomblé*, particularly with respect to the initiation rituals. For Vogel and collaborators [11], the market is a mandatory place of pilgrimage for the initiated in *Candomblé*. There, they can obtain all the materials that are necessary for the performance of rituals: necklaces, bracelets, clay pots for offerings, and plants.

Arriving at the market, I realized that all this trade was generated by the *Candomblé* religious demand. There were several vendors of herbs. Later, I learned that I could not label them all “vendors”; some called themselves “doctors of roots”, and they were experts in what they were selling. That logic implied that there were sellers who did not know the plants and could deceive people, and this often happened.

I was thrilled with the spectacle of colors and flavors, with the crazy coming and going of people with their shopping lists: plants for purification baths, cleaning baths, and incense to bring luck and ward off bad luck. I approached the first vendor and I was immediately turned away; I could barely introduce myself, much less ask about the plants. This was repeated in other failed attempts. Years later, I would return to this matter of the difficulty of gathering information on markets [12]. By the end of the day, I realized that the dynamics of the market were also unusual because the sellers had difficulty giving me attention, because they needed to attend to their customers, or because, as they clearly said, they believed I wanted to learn so that I could compete with them. Discouraged, I sat beside an old lady, a plant vendor with a very modest little shop. She approached me and said into my ear, “Take a bath with this little plant here and that sadness will go away”.

From there the conversation took off and I was learning about the plants, noting what I could and learning valuable lessons. I learned that information may vary from informant to informant; they showed me different plants with the same name. No, I was not mistaken. According to my old friend, “One of them was trying to trick me”. That is how, little by little, more than understanding the connection between the market and *Candomblé*, I was learning valuable lessons about collecting ethnobiological data. Even without anyone telling me, I took with me what could be called a prototype of a field notebook. The notes I made were very valuable to me later (something I never expected to happen), both to guide my future students and to propose research studies. Despite the impact it had on me at the time, then 21 years old and calling myself a marine biologist, I would not stop long to question this experience.

## Facing the unknown: the challenges of participant observation

I was a “theoretical” participant observer, so to speak. I read what I could about the subject. I holed up for hours in the library of the Faculty of Humanities to read everything I could before starting the job. Only now can I describe how I felt when I went to the *terreiros* for the first time to participate in the rituals, perhaps something very similar to what Bronislaw Malinowski narrates in his book-diary *Argonauts of the Western Pacific*[13]:

Imagine yourself, the reader, alone, surrounded only by your equipment on a tropical beach next to a native village, watching the launch or the boat that brought you departing to the sea until it disappeared from view. Let us suppose further that you are just a beginner with no experience, no script and no one who can help you. This describes exactly my initiation in field research on the south coast of New Guinea.

Imagine then yourself, the reader, in sole possession of one book, an old camera (almost disposable), and an almost atavistic fear generated by centuries of prejudice and religious persecution. This describes exactly my feelings and my apprehension. I was not on a desert island isolated from the modern world. Quite the contrary, I was in an urban area, a few meters from a bus stop. Nevertheless, upon entering the *terreiro*, I was transported to another universe. People sometimes wearing white clothes, sometimes colorful, women with head wraps and ample skirts. I arrived early in the morning because I was invited to witness parts of the initiation rites of a woman who was preparing herself to become a true follower. Invariably in such circumstances, ritual sacrifice occurs, and I witnessed two: the sacrifices of a goat and a rooster. It was unlike anything I expected, breaking down any negative bias. The animals were treated with deep respect and piety, and the sacrifices were performed quickly; I almost had the impression that the animals let themselves immolate voluntarily. The connection between the ritual sacrifice and the idea of medicine (the search for health) was reflected in my impressions. Later, I learned [14] the following myth that strengthened my initial impression:

There was a rivalry between *Orunmila* and *Osanyin*, who disputed the hierarchical superiority. *Olodumaré* then proposed a test in which Sacrifice and Medicine, sons of *Orunmila* and *Osanyin*, would be buried for seven days without food or drink, and the one whose son answered the call of his father at the end of that period would win the dispute. However, Sacrifice was aided by a rabbit who supplied him with food while he remained buried. Medicine, in turn, using his skills, dug a tunnel that led him to Sacrifice. He fed him in exchange for his silence. Thus, *Orunmila* won the contest, having been acknowledged as a power greater than that of *Osanyin*. The lesson is that if the sacrifice is done correctly, the man will not become ill, and therefore he will not have the need for remedies. This would explain why, very often, the *babalaô*, besides the advice given to the person, speaks of the need for sacrifice as well as for medicine.

Parts of the animals I saw being sacrificed were used to arrange the offerings to the gods, and the rest was cooked and offered to the people in a communal meal. The anthropologist Roberto Motta [15] speaks about the importance of the sacrifices from the social point of view: meat provides the community with animal protein. I realized how entering the field with presuppositions can prevent us from seeing the uniqueness and richness of the manifestations of the “other”. This person so similar to me in his/her wishes, in the will to live and conquer things, but yet so different in how he/she views the world. It was another lesson that I kept, and I thought to myself, if one day I return to do this kind of work, I will try to be more flexible in my experience with others so that my bias does not blind me.

The night was falling while all worked quickly on the preparations for the ceremony about to start. The ritual would be a celebration to honor the deity *Shango* (god of lightning and justice), King of *Koso*, syncretized with St. John the Baptist of Catholicism. Syncretism can be regarded as the “mix” of African religious nuances with Catholicism. Everything was ready. The *Yalorisha* (the *Mãe de Santo*, the “mother of the saint” or head of the *terreiro*) started the work by singing a few verses in the *Yorùbá* language, while behind her, some devotees played drums with a rhythmic sound. The other devotees, the *filhos de santo* (“sons of the saint”), danced in a circle following the verses and rocking their bodies with almost standardized movements.

This was followed by several songs until, at a certain moment, I realized that the rhythm of the drums was becoming more frantic and the followers were beginning to dance faster. I felt that there was a different atmosphere permeating the environment, and it culminated when the *Yalorisha* bent violently backward and forward with jerky movements that led her to the ground. When she stood up, with her hands hidden behind her back and with a heavy wheezing gasp, I realized she was in a trance, a trance of *Shango*. People began to applaud his arrival with clear devotion and enthusiasm. It was surprising to see a petite woman dancing nonstop for minutes, as if possessed by a hurricane. “*Shango* is exactly like this”, someone by my side said to me.

I was afraid when the *Yalorisha* approached me in a *Sango* trance and greeted me in a truculent way. However, I remembered, “*Shango* is exactly like this”. Despite being amazed at the time, this situation was not the biggest cultural shock I went through during the participant observation. *Shango* is a deity who has among his favorite meat dishes cooked rooster, *amalá* (a kind of crumbly food) made with an okra stew^g^. I hate the combination of these. When the time came to serve those present with the favorite food of *Shango*, I hid behind the crowd that accumulated for the communal meal. I was soon discovered and asked to be the first to be served. I cupped my hands, which served as a dish to receive the preferred food of *Shango*, the reason for fights narrated in the myths of this and other deities. I quickly devoured it as a strategy to shorten my suffering. However, I was immediately misunderstood, and someone shouted, “Serve the boy again; he liked the food”. Considering the respect for the ritual and the sacred manner with which each ingredient was included in the food, I had to accept the gift because I was afraid of offending my hosts; but this time, I ate it very slowly.

About Shango, wrote Verger [16] in *African Legends of the Orishas*:

Kawo Kabiyesi le!

Shango was the son of Oraniyan, the valiant warrior

Whose body was black on the right side and white on the left.

Brave man on the right.

Brave man on the left

Brave man at home,

Brave man in war.

Oraniyan was the founder of the Kingdom of Oyo in Yorubaland.

When he went to war, he always passed through Empe,

In Tapa Territory, which is also called Nupe.

Elempe, the king of that realm, formed an alliance with Oraniyan

And gave him his daughter in marriage.

This union produced a strong and vigorous son named Shango.

As a child in Tapa, Shango was always getting into trouble.

He was short-tempered, impatient, bossy,

And didn’t take kindly to any complaints.

Shango’s only pastimes were war games and fights.

(…)

The first place Shango visited called Koso.

When he arrived, the terrified townspeople asked:

“Who is this dangerous character?”

“He is so brutal and petulant!”

(…)

But Shango threatened them with his oshe.

His breath turned to fire and he destroyed a few houses with his lightning stones.

Everyone in Koso ran and begged for mercy, crying:

Kabiyesi Shango, Kawo Kabiyesi Shango Oba Koso!

“Let us all see and hail Shango, King of Koso!”

(…)

## Back to the origins

After these experiences, I went back to marine biology believing that my short ethnobiology career was over. However, through unexpected circumstances, I ended up writing a monograph on the subject, which helped me get a bachelor’s degree in biological sciences. During the defense of my thesis, I was often asked how I obtained some observations, as many of them are subject to secrecy. I countered with the argument that the participant observation brought me within close reach of my sources. Today, I think it was perhaps my innocence and those people’s desire to instruct me in some of their knowledge that made all the difference. During the fieldwork, I also learned that some things that I had said and heard could not be disclosed. I agreed to this deal with my helpful sources, and I disclosed only what was allowed. Maybe I had gradually gained the necessary confidence when I realized that my academic work could not be above the wishes of that group of people.

When I finally accepted myself as an ethnobiologist and, from the monograph, wrote a book called *Holy Leaves*, I felt the need to return to the places I had visited to leave some copies with my friends. I had a concern that Ferretti [17] translated very well:

Many *Pai de Santo*^h^ are avid readers of anthropology theses (…). Anthropology contributes, therefore, to the development and dissemination of knowledge in this way.

I agree with Ferretti, but many times during my later trips, I saw colleagues collecting information from respondents who were reproducing information read in anthropology books. This is supported by the words of Roberto Motta [18] when he says, “There is in the Afro-Brazilian religion a constant back and forth between rites and research, each influencing the other”. Again, the ethnobiologist neophyte began to realize that the anthropology method books talk about how our presence can affect data collection until people become accustomed to it, but we rarely hear how what we write affects how these people perceive themselves.

Many years later, I turned my attention to other topics of ethnobiological interest, and Afro-Brazilian cults came to represent my first unusual experience^i^. A short time ago, at the invitation of a friend and graduate student, I attended a *Candomblé* ritual at an important house that maintains this unique tradition in the region. It was the feast (ceremony) of *Yansan*. Upon entering, I was greeted by the priests of the house, who immediately alerted me to a flagrant religious transgression: I was wearing a black shirt, and that color is taboo because it symbolizes negativity. Still, out of consideration for me, they let me witness the ritual … People started to go into a trance of *Shango* … Jarring movements … Possessed by a hurricane. A person in a trance of the King of *Koso* approaches me like an old acquaintance, suddenly hugs me as if we are old friends who have not spoken to each other in a long time… Moves away a few feet and points to my shirt, with a visible air of aggravation and disapproval. I could only think of one thing: “I know, my friend, I’m sorry. I have to review the lessons”.

## Endnotes

^a^Systematic of Phanerogams is part of the botany that deals with the classification of so-called “flowering plants”(= phanerogams).

^b^Getúlio Vargas was the president of Brazil from 1930 to 1945 and from 1951 to 1954. Religious persecution which took place in his government was not only directed to the Candomblé, but also to other non-official religions practitioners, such as Kardecism - spiritualist religion founded in France.

^c^Nina Rodrigues was an intellectual of great influence. In addition to his vision of the trance, he defended the idea of white racial superiority. In a sense, this “racist” view was very common among intellectuals of the nineteenth century.

^d^It matches the plants in the genus *Ficus* both in Africa and Brazil. These plants are treated as sacred abode of a phytomorphic deity called Iroko.

^e^The word Nagô usually means the African who spoke the Yorubá language.

^f^Basically, the movie dealt with the religious-magical issue in a fantastic and sensationalist way. Wade Davis’s book offers a completely different view of such practices, contextualizing the historical and anthropological point of view. See Albuquerque et al. [19] to a recent review about the book.

^g^Scientific Name: *Abelmonchus esculentus*.

^h^“Pai de Santo” is also the name given to the babalorisha in Brazil.

^i^At the Masters, I decided to study the plants of the genus *Ocimum* given its importance in the african-Brazilian cults. As at the time, ethnobotany was not well accepted in academia (at least in Brazil), I have included in our study a strong component of classical botany.

## Competing interests

The author declares that he has no competing interests.
